# The Impact of Alveolar Recruitment Strategies on Perioperative Outcomes in Obese Patients Undergoing Major Gynecologic Cancer Surgeries: A Prospective Randomized Controlled Trial

**DOI:** 10.3390/diagnostics15111428

**Published:** 2025-06-04

**Authors:** Duygu Akyol, Funda Gümüş Özcan

**Affiliations:** Department of Anesthesiology and Reanimation, Başakşehir Çam and Sakura City Hospital, Republic of Turkey Ministry of Health, Basaksehir Olympic Boulevard Road, 34480 Basaksehir, Turkey

**Keywords:** alveolar recruitment strategy, lung-protective ventilation, obesity, postoperative pulmonary complications

## Abstract

**Background/Objectives**: Lung-protective ventilation (LPV) reduces postoperative pulmonary complications (PPCs) in obese patients. While the roles of low tidal volume and positive end-expiratory pressure (PEEP) in LPV have been established in patients with healthy lungs, the protective effect of alveolar recruitment strategies (ARSs) remains a subject of debate. This study aims to evaluate the benefit of ARSs in patients with low-to-moderate risk according to the Assess Respiratory Risk in Surgical Patients in Catalonia (ARISCAT) score undergoing gynecologic cancer surgery with LPV and low tidal volume intraoperatively. **Methods**: A total of 88 obese patients were evaluated in this study. They were divided into two groups as the non-ARS group (non-ARS) and the ARS group (ARS). Intraoperative hemodynamics, blood gas analyses, respiratory mechanics, mechanical ventilator parameters, and postoperative outcomes were compared in these obese patients. **Results**: A total of 40 obese patients undergoing major gynecological cancer surgery were included in this study. Although the non-ARS group presented with higher weight (*p* < 0.05), body mass indexes were similar to the ARS group. Intraoperative blood gas analysis revealed higher end-tidal carbon dioxide (etCO_2_) levels in the non-ARS group during the T2 and T3 time intervals (*p* < 0.05). In the ARS group, peak inspiratory pressure (PIP) at T3 was lower, while drive pressures at T1 and T2 and dynamic compliance at T3 were higher (*p* < 0.05). Radiologic atelectasis scores were higher in the non-ARS group, indicating more atelectatic lung images (*p* < 0.05). PPC rates were similar across both groups. **Conclusions**: Although the ARS demonstrated positive effects on lung mechanics and radiologic atelectasis scores in major open gynecologic cancer surgeries, it did not effectively reduce postoperative pulmonary complications.

## 1. Introduction

Obesity is a global health issue resulting from physical inactivity and poor nutrition related to lifestyle [1]. Alongside various systemic effects, it imposes significant stress on the respiratory system, reducing respiratory mechanics (vital capacity, inspiratory capacity, expiratory reserve volume, and functional residual capacity), which predisposes individuals to atelectasis [2]. Major gynecologic cancer surgeries are inherently associated with complications due to patient comorbidities and the extent of surgical interventions, complicating perioperative management. Perioperative anesthesia management significantly influences the development of postoperative pulmonary complications (PPCs), subsequently increasing hospital mortality and the length of stay [3,4]. The induction of general anesthesia affects gas exchange and respiratory mechanics, leading to decreased lung volumes and potential atelectasis [5].

The laparotomy approach required for surgeries in obese patients, coupled with lithotomy and Trendelenburg positioning, further complicates respiratory mechanics [6]. With the addition of the Trendelenburg position to lithotomy, airway pressure increases with the upward elevation of the intestinal contents and diaphragm, while functional residual capacity and lung compliance decrease. This makes respiratory mechanics difficult [7]. Therefore, to improve postoperative outcomes associated with general anesthesia, intraoperative lung-protective ventilation (LPV) strategies are increasingly employed [8]. Although the importance of tidal volume and PEEP has been established in LPV, the specific application and significance of alveolar recruitment strategies (ARSs) remain undetermined [9,10]. Our study is designed to emphasize the significance of the ARS in conjunction with intraoperative LPV in obese patients undergoing gynecologic cancer surgery, hypothesizing that it may enhance postoperative pulmonary outcomes.

## 2. Materials and Methods

**Figure 1:**
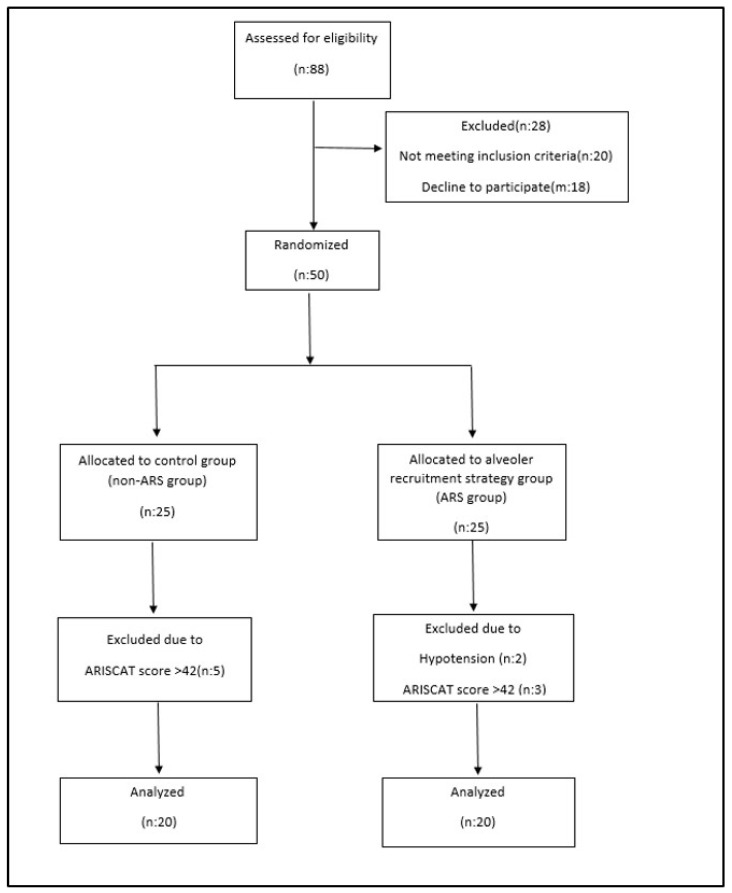
Consort flow diagram.

After approval by the Local Ethics Committee of Başakşehir Çam and Sakura City Hospital (approval no: 2023-583, approval date 22 November 2023), 68 patients who underwent major open gynecological cancer surgery for endometrial or ovarian cancer between 1 January 2024 and 1 April 2024 were evaluated. This study was registered at clinicaltrials.gov (study registration: NCT06619626, registration date: 10 May 2024). This study was conducted following the Helsinki Declaration and the principles of good clinical practice. Informed consent was obtained from all the participants. Patients with an American Society of Anesthesiologists (ASA) risk score of II or III, a body mass index (BMI) of 30–40 kg/m^2^, and an ARISCAT risk score of 26–44, who were scheduled for extubation in the operating room, were included. The ARISCAT risk score is a seven-variable model (age, preoperative oxygen saturation (SpO_2_), respiratory infection within a month prior to surgery, preoperative anemia, surgical incision, duration of surgery, and urgency of the procedure) that classifies patients into low-, moderate-, and high-risk groups [11]. Patients with severe cardiac disease (New York Heart Association class III or IV), respiratory disease (chronic obstructive pulmonary disease, recent respiratory infection, or ARISCAT risk scores > 44), BMI > 40 kg/m^2^, hypersensitivity or allergic reactions to any medication administered during the procedure, and patients ventilated for reasons other than abdominal surgery (e.g., acute respiratory distress syndrome or acute hypoxemic respiratory failure) were excluded. Respiratory mechanics become more difficult when morbid obesity (BMI > 40 kg/m^2^) is involved. Since oxygen saturation may be lower in these patients, this may increase the ARISCAT risk score, and higher values for respiratory complications may be obtained. Therefore, these patients were excluded from the study. Patients were randomized into two groups in a 1:1 ratio using the MedCalc 18.2.1 electronic software: No. 1 was designated by the software as non-ARS. No. 2 was designated as the ARS group. Patients were assigned into these groups (Figure 1).

All patients underwent standard ASA criteria monitoring (heart rate (HR), noninvasive mean arterial pressure (MAP), peripheral oxygen saturation (SpO_2_), and temperature), along with near-infrared spectroscopy (NIRS) for cerebral oxygenation and the patient state index (PSI, Masimo Corporation, Radical 7, Irvine, CA, USA) for the depth of anesthesia during the T1 interval. Routine induction of anesthesia (fentanyl 2–3 mcg/kg, propofol 1 mg/kg, and rocuronium 0.6 mg/kg) was followed by tracheal intubation (T2). Anesthesia maintenance was achieved with remifentanil infusion (0.05–0.2 mcg/kg/min) and sevoflurane inhalation (minimum alveolar concentration (MAC) of (0.6–0.9)). The depth of anesthesia was monitored via PSI within the 25–50 range. After induction, continuous arterial blood pressure monitoring and arterial blood sampling were conducted using a radial artery catheter. The ideal body weight was calculated using defined formulas (The predicted body weight of female patients was calculated as equal to 45.5 + 0.91 (centimeters of height—152.4)). Mechanical ventilation was initiated in volume-controlled ventilation (VCV) mode with tidal volumes adjusted to 6–8 mL/kg based on the ideal body weight, 8 cmH_2_O PEEP, and respiration rates were adjusted to maintain an end-tidal carbon dioxide (etCO_2_) range of 35–45 mmHg. All patients received low-flow anesthesia (flow: 0.25–1 L). To protect patients from hypoxia, inspired oxygen pressure alarm limits were set at >30 mmHg; FiO_2_ and flow parameters in low-flow anesthesia were adjusted according to arterial blood gas PaO_2_ values and NIRS readings.

**Figure 2:**
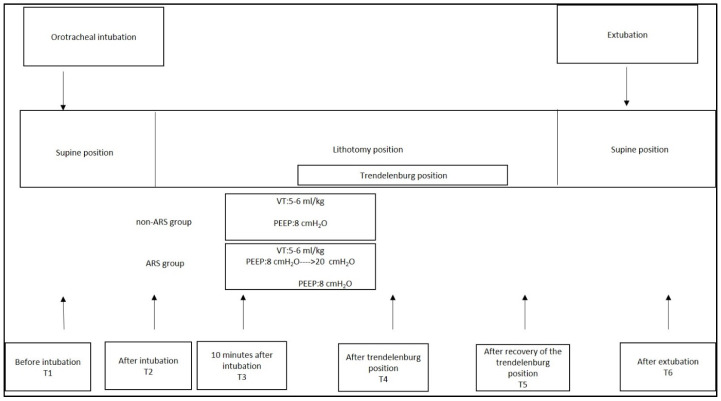
Study protocol of the groups.

Mechanical ventilator parameters (TV, FiO_2_, flow, peak inspiratory pressure (PIP), plateau pressure (Pplato), etCO_2_, and dynamic compliance (Cdyn)) and hemodynamic, cerebral oxygenation, and arterial blood gas parameters (pH, partial carbon dioxide pressure (pCO_2_), partial oxygen pressure (pO_2_), ctO_2C_, and lactate) were recorded after orotracheal intubation (T2) and ten minutes post ventilation (T3). After ten minutes of ventilation, patients assigned to undergo ARS were switched to pressure-controlled ventilation (PCV) mode, and ARS commenced. PIP was escalated from 30 cmH_2_O, and PEEP increased in increments of 2 until it reached 20 cmH_2_O, with ventilation maintained for 2 min at each PEEP change. Respiratory rates were adjusted to maintain PaCO_2_ at 35–45 mmHg. If the mean arterial pressure dropped by more than 20% or if systolic blood pressure fell below 90 mmHg, ARS was discontinued, and those patients were excluded from the study. Once 20 cmH_2_O PEEP was reached, PEEP was reduced back to 8 cmH_2_O, and mechanical ventilation resumed in VCV mode. All patients were positioned in lithotomy for ease of surgical access, followed by a 20° Trendelenburg position after surgical incision. Mechanical ventilation parameters, blood gas parameters, and hemodynamic parameters were recorded following Trendelenburg positioning (T4) and after correcting the position (T5). Intraoperative fluid management included targeted fluid therapy using non-invasive advanced hemodynamic monitoring. Hemodynamic parameters of extubated patients were recorded post-extubation (T6) (Figure 2). Fluid administration, pain management, vasoactive medications, and blood transfusions were managed according to routine protocols. Patients with a modified Aldrete score of > 9 were discharged to the ward. On postoperative day 1, routine X-ray imaging was performed and evaluated according to the radiological atelectasis score (0, clear lung field; 1, plate-like atelectasis or slight infiltration; 2, partial atelectasis; 3, lobar atelectasis; and 4, bilateral lobar atelectasis) [12]. Postoperative pulmonary complications were determined using a sliding scale from 1 to 4, with increasing levels representing the severity of the worsening complication (grade 1: microatelectasis; grade 2: bronchospasm, hypoxemia, and atelectasis; grade 3: pleural effusion, pneumonia, and pneumothorax; and grade 4: respiratory failure) [13]. Postoperative assessments recorded pulmonary and non-pulmonary complications (surgical complications (anastomotic leakage and need for surgical re-intervention), systemic inflammatory response syndrome (SIRS) and sepsis, wound infections, hepatic complications, and renal complications), patients requiring hospitalization, mechanical ventilation status, and the length of stay. One anesthetist performed intraoperative management, while another assessed postoperative lung images, scores, and outcomes. The anesthetist assessing postoperative outcomes was blinded to the study groups.

### 2.1. Sample Size Calculation

Based on power analysis predicting the effect of the primary objective on postoperative pulmonary complication scores, the minimum sample size was set at 20 for each group, with an effect size of 1.2, a 5% margin of error, and 95% power. 

### 2.2. Statistical Analysis

Statistical analyses were performed using NCSS 11 (Number Cruncher Statistical System, 2017 Statistical Software). Frequency and percentage values were presented for categorical variables. Continuous variables were reported as the mean ± standard deviation and the median (IQR). The normality of continuous variables was assessed using the Kolmogorov–Smirnov test. Chi-square tests were employed to evaluate relationships between categorical variables. Fisher’s exact test and Fisher–Freeman–Halton tests were utilized as appropriate. Independent sample *t*-tests were conducted to compare means between two independent groups with normally distributed continuous variables. The Mann–Whitney U test was employed for comparisons between two independent groups that did not meet normal distribution assumptions. The analysis of variance test (post hoc: Bonferroni correction) was used for the group comparison of the normally distributed variables, and the Kruskal–Wallis *H* test (post hoc: Dunn’s correction) was used for the intergroup comparison of the non-normally distributed variables. The chi-square and Fisher exact tests were used for the intergroup comparison of the categorical variables. The significance level was set at α = 0.05. *p* < 0.05 was determined as statistically significant.

## 3. Results

In this study that evaluated a total of 40 patients undergoing gynecologic cancer surgery—20 in the non-ARS group and 20 in the ARS group—the mean ages of the patients were 56.050 ± 9.411 and 55.200 ± 12.614, respectively (*p* > 0.05). While the weights of patients in the non-ARS group were statistically significantly higher (*p* < 0.05), the body mass indices (BMIs) were comparable between both groups. When ARISCAT scores were assessed, the median (IQR) values for the non-ARS and ARS groups were 41 (37.25–41) and 36 (34–41), respectively, demonstrating similarity. In the non-ARS group, 70% was classified as ASA II, while 90% of the ARS group was classified as ASA II (*p* < 0.05). Lengths of hospital stay and rates of admission to intensive care were comparable between both groups (*p* > 0.05) (Table 1).

**Figure 3:**
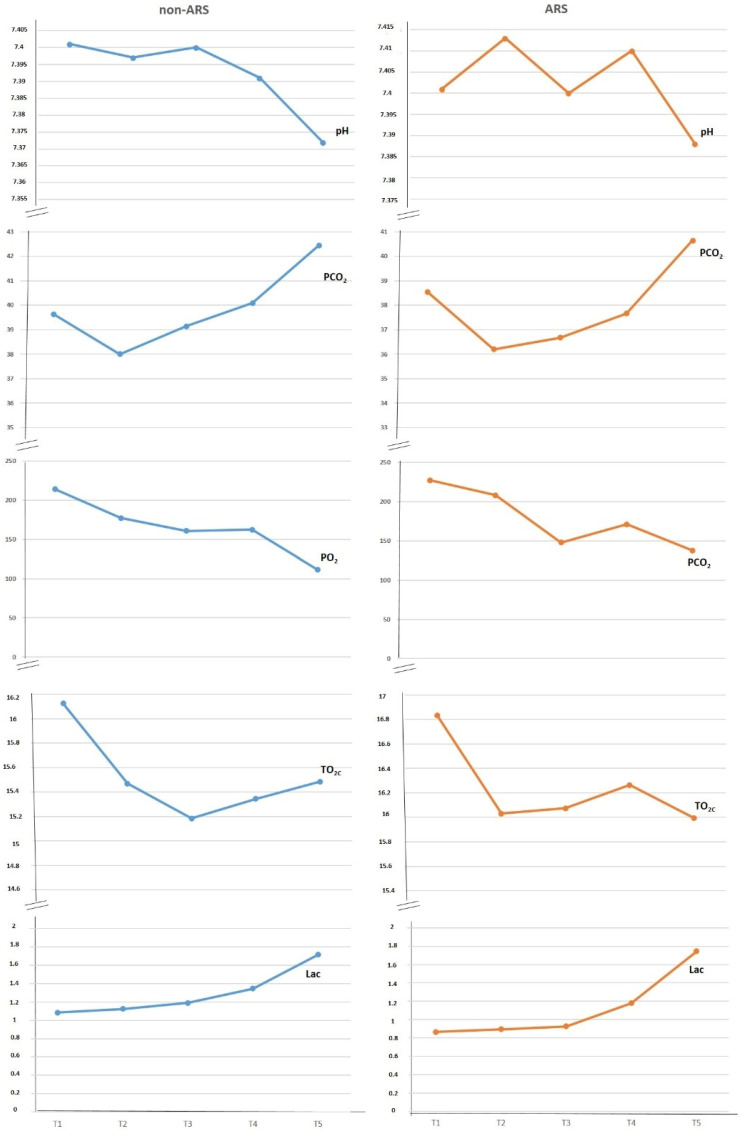
Evaluation of blood gases between groups.

The evaluated blood gas parameters, including pH, pO_2_, ctO_2c_, and lactate, showed no significant differences between the groups during T2, T3, T4, T5, and T6. However, etCO_2_ values were higher in the non-ARS group compared to the ARS group during T3 and T4 (Figure 3).

Hemodynamic parameters and cerebral oxygenation values for both the non-ARS and ARS groups are detailed in Table 2. The patients’ KTA, MAP, and SpO_2_ values at time points T1, T2, T3, T4, T5, and T6 were found to be similar across both groups. When central oxygenation values (NIRS_right_ and NIRS_left_) were analyzed, no statistically significant difference was observed between the two groups.

Mechanical ventilator parameters are summarized in Table 3. Tidal volume, frequency, FiO_2_, and flow rates were similar in both groups. Assessing PIP revealed lower pressures in the ARS group during T4 (*p* < 0.05). Although plateau pressures were higher in the ARS group post-intubation (*p* < 0.05), following the application of ARS and the Trendelenburg position, pressures were similar to those in the non-ARS group during these intervals. Driving pressures (DPs) were higher in the ARS group during the T2 and T3 time intervals (*p* < 0.05). However, since lung-protective ventilation was maintained during all time intervals, driving pressures remained below 15 cmH_2_O. Dynamic compliance (Cdyn) was higher in the ARS group compared to the non-ARS group at T4 (*p* < 0.05) (Table 3).

When examined for radiologic atelectasis scores, atelectasis was not observed in 25% of the non-ARS group and 50% of the ARS group. In the non-ARS group, Grade 1 was identified in nine patients, Grade 2 in two patients, Grade 3 in two patients, and Grade 4 in two patients. In contrast, the ARS group exhibited Grade 1 in nine patients, with one patient showing Grade 4. No patients in the ARS group presented with Grade 2 or 3 (*p* < 0.05). PPC rates were comparable between both groups (Table 4).

Extrapulmonary complications were similar among both groups, with SIRS and sepsis at 0.1, surgical complications at 1.0, surgical site infections at 0.1, and renal complications at 1.2 in the non-ARS and ARS groups, respectively (Table 4).

## 4. Discussion

Our findings indicate that within the intraoperative lung-protective ventilation strategy for obese patients, the inclusion of alveolar recruitment strategies resulted in lower peak inspiratory pressures and improved dynamic compliance, leading to reduced radiological atelectasis scores, with 50% of patients experiencing no atelectasis. However, this did not translate into a reduction in clinically significant postoperative pulmonary complications as reflected by the graded scales. Despite higher pCO_2_ levels observed in the control group compared to the ARS group during T2 and T3, these values remained within normal ranges for both groups, indicating no clinical significance. Abdominal surgeries carry a high and unavoidable risk of postoperative pulmonary complications [3,14,15]. Additionally, prolonged surgical duration exceeding three hours is associated with deteriorations in postoperative pulmonary function [16]. The development of atelectasis due to general anesthesia adversely affects mechanical ventilation dynamics and can contribute to lung injury [17]. Strategies employing low tidal volumes, moderate PEEP, and recruitment maneuvers in lung-protective ventilation may enhance postoperative pulmonary outcomes in high-risk abdominal surgeries [18]. While the implementation of low tidal volume and PEEP has shown survival benefits, the impact of intraoperative LPV in patients without underlying lung disease is still debated. The exacerbating factor of obesity complicates the situation, as increased adipose tissue leads to reduced total lung capacity and functional residual capacity, resulting in impaired respiratory mechanics. It does this by decreasing lung compliance and tidal volumes and impairing alveolar oxygenation due to increased abdominal and intrathoracic pressure in obese patients and the effects of general anesthesia [19]. Even the ventilation strategy and postoperative pulmonary complications have been associated with the length of hospital stay and mortality in obese patients [20]. It is known that the level of PEEP is influenced by chest wall compliance and the body mass index, with individual optimal PEEP values varying [21]. To mitigate this, LPV is implemented with low tidal volumes and moderate PEEP levels (5–8 cmH_2_O) to prevent atelectasis while minimizing hemodynamic instability and fluid infusion needs [22,23]. In a meta-analysis, it was shown that there were large differences in intraoperative ventilation strategies in preventing perioperative pulmonary complications in obese patients [24]. One study even showed that high PEEP (12 cmH_2_O) did not reduce postoperative complications compared to low PEEP (0–2 cmH_2_O) in abdominal operations, leaving the role of PEEP in postoperative outcomes unclear [23]. Furthermore, driving pressures below 15 cmH_2_O are essential for establishing lung-protective ventilation alongside low tidal volumes and moderate PEEP [25]. Dynamic compliancy provides alveolar stability and improves intraoperative oxygenation by decreasing the end-expiratory respiratory volume closer to the functional residual capacity by decreasing the drive pressure with PEEP titration [26]. In our patients, tidal volume was determined based on the ideal body weight and delivered with moderate PEEP while maintaining driving pressures below 15 cmH_2_O. Despite the improved alveolar recruitment with ARS, the overall clinical significance in postoperative hospital and intensive care unit settings remained minimal [27].

Numerous ARS methods have been proposed, yet a gradual recruitment maneuver is preferred to avoid excessive alveolar distension [28]. Although ARS has been applied through continuous high inspiratory pressures or incremental PEEP increases, an optimal technique remains unattained, and the timing and methods of intraoperative application are not standardized. Despite evidence demonstrating improvements in intraoperative oxygenation associated with alveolar recruitment maneuvers, the recurrence of atelectasis within 40 min post-maneuver and the lack of long-term effect have been noted [29,30]. Therefore, ARS appears to be more effective when integrated within LPV frameworks [31]. In a meta-analysis evaluating ventilation strategies in obese patients, the combination of PEEP and ARS was found to possess the highest potential for reducing postoperative atelectasis in volume-controlled modes [32]. High PEEP and ARS reduce postoperative atelectasis, reduce drive pressure, homogenize ventilation, and improve postoperative pulmonary outcomes but may increase inflammation and impair hemodynamics and lymphatic drainage [33].

Nonetheless, ARS can result in increased intrathoracic pressure and a decrease in left ventricular diastolic end pressure and cardiac output, leading to hypotension [23]. Thus, we advocate for a carefully controlled hemodynamic approach for the incorporation of recruitment maneuvers as part of lung-protective ventilation, given their potential to improve intraoperative lung mechanics and reduce postoperative atelectasis through the maintenance of low driving pressures.

The lack of impact from ARS on alveolar oxygenation, alongside diverse results ranging from no effect to hemodynamic instability, has been observed [34]. According to systematic reviews, intraoperative pO_2_ values were consistently higher in groups undergoing ARS compared to the control [35]. In obese patients, meta-analysis results indicate that ARS improves intraoperative arterial oxygenation [24]. However, in our study, comparable arterial oxygenation levels may be attributed to adjustments in low-flow anesthesia based on arterial blood gas results rather than fixed flow rates.

The limitations of this study include the inability to generalize results to laparoscopic cases. Additionally, patients with a BMI exceeding 40 kg/m^2^, who are more susceptible to respiratory impairments, were excluded. Furthermore, postoperative complications were evaluated only in the short term by chest radiography and PPC grading scales. For the postoperative pulmonary evaluation, results can be evaluated with studies that can be combined with tomography or lung ultrasonography. Long-term outcomes were not evaluated.

## 5. Conclusions

The application of intraoperative lung-protective ventilation in obese patients remains uncertain. Although its application in the intraoperative period is somewhat controversial, the feasibility of lung-protective ventilation strategies in obese patients (BMI: 30–40 kg/m^2^) in gynecological open cancer surgeries and the potential enhancement of dynamic compliance and PIP through the use of intraoperative ARS have been demonstrated. However, additional studies are warranted to reinforce the beneficial effects of ventilation strategies, identify optimal PEEP levels, determine the best alveolar recruitment methods, and explore their impacts on clinical outcomes.

## Figures and Tables

**Table 1:** Basic characteristics of the non-ARS and ARS group patients.

	Non-ARS (*n*:20)	ARS (*n*:20)	*p*
Age, years	56.050 ± 9.411	55.200 ± 12.614	0.810
Weight, kg	88.750 ± 9.513	82.500 ± 5.145	0.015 *
Height, cm	160.100 ± 5.025	158.150 ± 6.301	0.286
BMI, kg/m^2^	34.705 ± 2.863	33.040 ± 2.734	0.068
Duration of surgery, minutes	195.950 ± 37.888	234.850 ± 91.383	0.091
ARISCAT score	41 (37.25–41)	36 (34–41)	0.08
Comorbidity, *n* (%)			0.942
HT	8 (40)	7 (35)	
DM	4 (20)	5 (25)	
Lung disease	1 (10)	1 (5)	
Thyroid dysfunction	1 (10)	1 (5)	
Other diseases	4 (10)	1 (5)	
ASA, *n* (%)			0.118
2	14 (70)	18 (90)	
3	6 (30)	2 (10)	
Length of hospital stay, days	4.850 ± 1.225	4.900 ± 1.165	0.896
Presence of ICU hospitalization	9 (45)	5 (25)	0.190

ARISCAT, Assess Respiratory Risk in Surgical Patients in Catalonia; ASA, American Society of Anesthesiology; HT, hypertension, DM, diabetes mellitus; ICU, intensive care unit. * *p* < 0.05.

**Table 2:** Evaluation of hemodynamics and cerebral oxygenation between groups.

	Non-ARS (*n*:20)	ARS (*n*:20)	*p*
HR, beats/minute			
T1	82.550 ± 13.264	89.500 ± 17.831	0.170
T2	77.315 ± 15.975	84.800 ± 16.334	0.157
T3	72.200 ± 11.865	73.700 ± 12.818	0.730
T4	67.750 ± 13.054	74.850 ± 14.829	0.116
T5	70.900 ± 13.995	72.250 ± 13.186	0.755
T6	83.550 ± 18.740	89.150 ± 16.161	0.318
MAP, mmHg			
T1	107.250 ± 18.724	114.200 ± 17.887	0.237
T2	87.200 ± 16.093	94.300 ± 19.520	0.217
T3	86.600 ± 23.349	80.850 ± 22.850	0.436
T4	94.050 ± 22.312	91.700 ± 15.647	0.702
T5	85.800 ± 16.093	78.200 ± 12.297	0.102
T6	99.800 ± 20.106	94.450 ± 12.725	0.321
SpO_2_, %			
T1	99.150 ± 1.225	98.500 ± 1.791	0.189
T2	100 (99.25–97)	100 (99–100)	0.226
T3	99.300 ± 1.218	99.350 ± 1.089	0.892
T4	98.50 (97–100)	99 (98–100)	0.210
T5	100 (99–100)	99 (98.25–100)	0.408
T6	98.750 ± 1.446	99 ± 1.414	0.584
NIRS_right_			
T1	67.800 ± 5.745	71.650 ± 11.449	0.187
T2	69.100 ± 7.340	72 ± 10.130	0.306
T3	65.50 (60–70)	70 (64.25–73.50)	0.147
T4	69.50 (61.75–72)	68 (63.25–73.75)	0.924
T5	67.550 ± 7.472	69.800 ± 10.144	0.429
T6	70 (65.75–72.50)	72 (66.25–76)	0.284
NIRS_left_			
T1	70.650 ± 8.171	69.750 ± 8.018	0.727
T2	71.850 ± 8.317	70.300 ± 7.427	0.538
T3	70.750 ± 9.930	67.400 ± 7.535	0.237
T4	71.050 ± 10.262	66.300 ± 7.644	0.105
T5	72.150 ± 11.379	67.350 ± 8.336	0.136
T6	71.400 ± 9.996	70.500 ± 7.976	0.755

HR, heart rate; MAP mean arterial pressure; SpO_2_: oxygen saturation as measured by pulse oximetry, NIRS, near-infrared spectroscopy.

**Table 3:** Comparison of respiratory mechanics and mechanical ventilator parameters.

	T2		T3		T4		T5	
	Non-ARS	ARS	Non-ARS	ARS	Non-ARS	ARS	Non-ARS	ARS
TV mL	430.650 ± 39.349	433 ± 34.957	436.400 ± 24.469	434.80 ± 44.139	432 ± 27.028	447.100 ± 40.167	438.850 ± 29.205	460.900 ± 56.616
Respiratory rate	12 (12–12.75)	12 (12–12)	12.200 ± 0.410	12.450 ± 0.604	12.700 ± 1.031	12.750 ± 0.966	12.550 ± 0.944	12.850 ± 10.308
FiO_2_ %	60.750 ± 7.304	63.750 ± 5.590	59.500 ± 6.261	63.250 ± 4.94 *	62.250 ± 4.127	62.750 ± 3.79	61.750 ± 6.544	64.250 ± 5.447
Flow L	0.935 ± 0.142	0.895 ± 0.203	0.655 ± 0.238	0.535 ± 0.200	0.537 ± 0.206	0.520 ± 0.155	0.675 ± 0.244	0.552 ± 0.215
PIP cmH_2_O	18.00 ± 2.533	19.350 ± 2.084	18 (16.25–19.75)	19.50 (17.25–21.75)	22 (21–23.75) *	20 (18.25–22)	18.500 ± 2.982	18.800 ± 2.419
P_plato_ cmH_2_O	16.550 ± 3.235	19.050 ± 2.372 *	19 (16–21.50)	18 (15.25–18.75)	19.750 ± 2.291	19.200 ± 3.365	18.500 ± 2.982	18.800 ± 2.419
DP cmH_2_O	8.550 ± 3.235	11.150 ± 2.539 *	9.050 ± 2.665	11.500 ± 4.123 *	10.950 ± 3.546	11.650 ± 2.277	10.450 ± 2.946	10.650 ± 2.345
C_dyn_ mL/cmH_2_O	51.375 ± 9.012	56.490 ± 12.725	55.040 ± 10.770	53.475 ± 13.386	42.590 ± 7.796	53.445 ± 15.204 *	52.480 ± 12.536	55.480 ± 12.935

C_dyn_—dynamic compliance, DP—driving pressure, FiO_2_—fraction of inspired oxygen, P_plato_—plateau pressure, and TV—tidal volume. Data are presented as the mean ± sd or % as appropriate. * *p* < 0.05.

**Table 4:** Comparison of radiological atelectasis score and pulmonary and extrapulmonary complications between groups. PPC: postoperative pulmonary complication.

	Non-ARS (*n*:20)	ARS (*n*:20)	*p*
Radiological Atelectasis Score, *n* (%)			0.039 *
0	5 (25)	10 (50)	
1	9 (45)	9 (45)	
2	2 (10)	0	
3	2 (10)	0	
4	2 (10)	1 (5)	
Grade scale for PPC, *n* (%)			0.170
0	6 (30)	9 (45)	
1	10 (50)	10 (50)	
2	2 (10)	1 (5)	
3	2 (10)	0	
4	0	0	
Extrapulmonary complications, *n* (%)	2 (10)	4 (20)	0.360
SIRS Sepsis	0	1 (5)	
Surgical complication: Anastomotic leakage and need for surgical re-intervention	1 (5)	0	
Surgical site infection	0	1 (5)	
Renal complications	1 (5)	2 (10)	

* *p* < 0.05.

## Data Availability

Supporting data will be made available upon request.
